# The Massachusetts Veterinary Association

**Published:** 1893-02

**Authors:** Austin Peters

**Affiliations:** Secretary


*The Massachusetts Veterinary Association,*—The regular meeting of the Massachusetts Veterinary Association was held Wednesday evening, November 23d, 1892, at 19 Boylston Place, Boston.

The President, L. H. Howard, in the chair. Members present: Drs. Billings, Blackwood, Becket, Emerson, Harrington, Howard, Marshall, Osgood, Winchester, Parker, LaBaw, and the Secretary. Honorary member: Dr. Stickney.

The minutes of the last meeting were read and accepted, after Dr. Winchester had made the following corrections: That Dr. Bryden spoke of the outbreak in the Spring of 1873 as the "so-called outbreak of spinal meningitis;" and also that it was at Dr. Bryden's request that the name of the professor at the Harvard Medical School was withheld from publication, in connection with his remarks on springhalt.

*Unfinished Business:* Dr. Osgood reported that the springhalt horse was in such a state two days after our last meeting that he had him killed and sent to

Ward's, with the message that he would like the cord, but through some misunderstanding it was boiled out, and was therefore useless. A few days later a dog. with what appeared to have springhalt, was sent to the Harvard Veterinary Hospital. One hind leg was affected. Dr. Osgood sent this dog's cord to Mr. Bolton, of Clark University, instead of the horse's.

Dr. Stickney thought that the dog ought to have had an exostosis on the hock of the lame leg He had a pointer once himself with exostosis on the hock and springhalt Dr Howard read a letter from Mr Bolton in reply to one he wrote him asking about the publication of Mr. Bolton's paper on "Springhalt." Mr. Bolton said that his paper would appear in the January number of the *Journal of Mental and Nervous Diseases.*

*Reports of Committees:* Committee on Resolutions in regard to the new "National Veterinary School," reported through Dr. Winchester After remarks by Drs. Billings and Stickney, the resolutions were adopted on motion of Dr. Billings, seconded by Dr. Emerson. Moved by Dr. Billings, that copies of the resolutions adopted be sent to the Honorable Secretary of Agriculture, the Secretary of the so-called school, the JOURNAL OF COMPARATIVE MEDICINE, the *American Veterinary Review*, and the U. S. Committee on Agriculture, chairman from the Senate, Hon. A. S. Paddock, chairman from the House, Hon. W. H. Hatch. Seconded by Dr. Winchester. Carried.

*Admission of Members:* The name of Dr. W. M. Balmer balloted upon. Ten votes cast, all in the affirmative. The President therefore declared him elected to membership.

*New Business:* Dr. Osgood thinks that it is a farce to require applicants for membership to present their credentials to the Executive Committee, and that there is no need of its seeing a man's credentials, except we know nothing about him. He thinks the clause in the Constitution interferes with men joining who have their diplomas in frames, and perhaps fifty or one hundred miles away. Dr. Howard reads the clause in the Constitution, showing that nothing is said about diploma, but that only the term credentials is used. Dr. Osgood, as chairman of the Executive Committee, Drs. Emerson and Blackwood, members of the committee, agreeing with him, then gave notice that he reported favorably upon the names of Drs. G. B. Foss and S. O. Fowle as applicants for membershlp, although their diplomas had not been presented to the committee, and that he requires the Secretary to give notice that their names will be voted upon at the next meeting.

Dr. Billings then made a short speech, in which he refuted remarks made by Dr. Salmon about him, made at the meeting of the U. S. V. M. A., in Boston, last September. He also spoke of his work in the West upon Texas Fever, *Maladie du Coit*, "Corn Stalk Disease," and summer septicæmia of stock. Concluding, spoke of State and Government veterinary positions, and thought that one reason why they are not filled by better men were that the salaries were so low as only to procure the services of a cheap class of men.

*Reports of Cases:* Dr. Blackwood showed specimens of enlarged axillary and cardiac lymphatic glands from a horse which died, whose symptoms in life were simply tremendous swellings of the neck and chest, some of the glands being as large as kidneys. The subcutaneous cellular tissue of the neck was found to be about one and a half inches thick.

Dr. Stickney remembered once seeing a somewhat similar case, which died in less than twenty-four hours after the first appearance, and the same owner had two other cases afterward, but in this instance he thought it might have been due to some defect in the drainage.

Dr. Billings said, we have a lot of isolated cases of infectious diseases from bad drainage, etc., that have as yet not even been named.

Meeting then adjourned. AUSTIN PETERS, *Secretary.*

---

The regular meeting of the Massachusetts Veterinary Association was held at 19 Boylston Place, Boston, Wednesday evening, Dec. 28th, 1892.

President L. H. Howard in the chair. Members present: Drs. Bryden, Blackwood, Emerson, Harrington, Howard, Marshall, Winchester, Parker, LaBaw, Carlton, and the Secretary. Honorary member, Dr. Stickney. Visitor, Mr. Brown, a student at the Harvard Veterinary School.

Minutes of last meeting read and accepted.

*Admission of Members:* Names of S. O. Fowle, M.D.V. (Harv.), and G. B. Foss, M.D.V. (Harv.), balloted upon for membership. Nine votes were cast, all in the affirmative. The President, therefore, declared the candidates elected.

*Applications for Membership:* The applications of Dr. C. R. Simpson, of Somerville, and Dr. T. W. Shaw, of Springfield, for membership in the Massachusetts Veterinary Association, were reported favorably upon by the Executive Committee. Their names were laid upon the table to be balloted upon at the next regular meeting, under the rules.

*New Business:* Dr. Winchester thinks that the requirements of the Constitution providing for essays at these meetings should be lived up to. The President says that there is no clause in the Constitution providing for essays. They are voluntary contributions. Dr. Winchester says, then the fact of our not having any lately must be due to a lack of ardor on the part of the officers of the Association.

This accusation is indignantly denied by the officers.

Dr. LaBaw then volunteers a paper for the January meeting upon "Punctured Wounds of the Feet."

Dr. Blackwood offers to read at the February meeting upon "The Destruction of Microbes."

Dr. Parker offers a paper for the March meeting upon "Scouring in Calves."

Dr. Marshall offers a paper for the May meeting upon "The Production of Milk."

Dr. Carlton offers a paper for the meeting of December, 1893, upon "Homeopathy Applied to Veterinary Medicine."

Dr. Winchester then wished to know if the Association had any directions for its members in the Comitia Minora of the U. S. V. M. A., at the meeting soon to be held. The members of the Association did not have any special instructions to give at this time

Dr. Bryden then read a short but interesting paper upon "A Case of Seedy Toe." Vide fol. 69.

A general disussion followed, taken part in by Drs. Emerson, Winchester, Howard and Marshall. Dr. Winchester brings up the point, is this disease caused by a parasite? Dr. Bryden replies that he thinks the disease appears first, and furnished a favorable field for the development of the parasite.

Moved by Dr. Emerson that the essayist be given a vote of thanks. Seconded by Dr. LaBaw. Carried.

Meeting then adjourned. AUSTIN PETERS, *Secretary.*

